# Biomarkers in Spinal Cord Injury: Prognostic Insights and Future Potentials

**DOI:** 10.3389/fneur.2019.00027

**Published:** 2019-01-29

**Authors:** Ahmed A. Albayar, Abigail Roche, Przemyslaw Swiatkowski, Sarah Antar, Nouran Ouda, Eman Emara, Douglas H. Smith, Ali K. Ozturk, Basem I. Awad

**Affiliations:** ^1^Department of Neurosurgery, Penn Center for Brain Injury and Repair, University of Pennsylvania, Philadelphia, PA, United States; ^2^Department of Medical Biochemistry, Faculty of Medicine, Mansoura University, Mansoura, Egypt; ^3^Department of Neurosurgery, Faculty of Medicine, Mansoura University, Mansoura, Egypt

**Keywords:** spinal cord injury, biomarkers, central nervous system injury, neuroinflammation, proteomics, spinal cord injury pathophysiology

## Abstract

Spinal Cord Injury (SCI) is a major challenge in Neurotrauma research. Complex pathophysiological processes take place immediately after the injury and later on as the chronic injury develops. Moreover, SCI is usually accompanied by traumatic injuries because the most common modality of injury is road traffic accidents and falls. Patients develop significant permanent neurological deficits that depend on the extent and the location of the injury itself and in time they develop further neurological and body changes that may risk their mere survival. In our review, we explored the recent updates with regards to SCI biomarkers. We observed two methods that may lead to the appearance of biomarkers for SCI. First, during the first few weeks following the injury the Blood Spinal Cord Barrier (BSCB) disruption that releases several neurologic structure components from the injured tissue. These components find their way to Cerebrospinal Fluid (CSF) and the systemic circulation. Also, as the injury develops several components of the pathological process are expressed or released such as in neuroinflammation, apoptosis, reactive oxygen species, and excitotoxicity sequences. Therefore, there is a growing interest in examining any correlations between these components and the degrees or the outcomes of the injury. Additionally, some of the candidate biomarkers are theorized to track the progressive changes of SCI which offers an insight on the patients' prognoses, potential-treatments-outcomes assessment, and monitoring the progression of the complications of chronic SCI such as Pressure Ulcers and urinary dysfunction. An extensive literature review was performed covering literature, published in English, until February 2018 using the Medline/PubMed database. Experimental and human studies were included and titles, PMID, publication year, authors, biomarkers studies, the method of validation, relationship to SCI pathophysiology, and concluded correlation were reported. Potential SCI biomarkers need further validation using clinical studies. The selection of the appropriate biomarker group should be made based on the stage of the injuries, the accompanying trauma and with regards to any surgical, or medical interference that might have been done. Additionally, we suggest testing multiple biomarkers related to the several pathological changes coinciding to offer a more precise prediction of the outcome.

## Introduction

Spinal Cord Injury (SCI) remains one of the most devastating and difficult to manage medical pathologies despite the tremendous progress in neuroscience and neurosurgery. The National SCI Statistical Center (NSCISC) in 2016 reported more than 17,000 new cases and a total of 282,000 patients living with SCI in the USA alone. This type of injury most commonly occurs in young to middle-aged populations due to road traffic accidents, violence, and contact sports (1). The survivors of SCI are left with immediate neurological losses and a state of spinal shock that jeopardizes continued their survival (2). Unfortunately, survivors of SCI often experience a severe decrease in quality of life, as they struggle with the burden of continuous medical care to manage SCI-related complications and comorbidities.

SCI is a broad term that includes variable grades of neurological deficits. Only about 1% of SCI patients experience injuries that fully resolve. Almost 45% of cases experience severe neurological loss, in some cases with complete or incomplete tetraplegia, with or without respiratory compromise (1). However, the available tools to assess the severity of spinal cord tissue destruction and to predict recovery for SCI patients are still limited, particularly in developing countries, because they often require lavish resources to perform various imaging tests to decide the best neurosurgical intervention for each case. It is also essential to address the lack of reliable treatment interventions for such groups of patients, as most of the medical decisions are targeting the stabilization of the patients and preventing further injury, but no definitive treatment for the present state of the Central Nervous System (CNS) trauma exists (3, 4).

The progression of SCI is divided into different stages/pathophysiological phases experienced by the patient. In the first stage, termed the acute stage or primary injury, the patient not only develops neurologic deficits directly related to the injury but also suffers from spinal shock in the form of respiratory/circulatory disruption and urinary incontinence. The primary attention in this stage is dedicated to protecting patient's airways, ensuring proper respiratory function, and providing hemodynamic support. Additionally, until recently became not recommended, administering high-dose steroids was also employed in the first 8 h management postinjury in order to minimize further neurological inflammation and deficits (5, 6). Within a few days to postinjury, the patient progresses to the subacute stage or the secondary phase, where they typically recover from the spinal shock. However, the neurologic deficits, as well as the accompanying complications of the original trauma, persist. Finally, the patient advances to the chronic phase of SCI, which is dependent upon the general state of the patient, and the extent of the injury (3). During this phase, the patients may show partial neurologic recovery, maturation of adaptive mechanisms, or the onset of more delayed neurologic symptoms such as neuropathic pain, dysautonomia, urinary bladder dysfunction, musculoskeletal atrophy, lipodystrophy, and abnormal skeletal postures (7). The clinical course of SCI is noticeably related to the pathophysiology of the injury. As the injury occurs, local ischemia and edema develop, resulting in further ionic dysregulation and mitochondrial dysfunction, ultimately leading to necrotic cell death of various cell populations in Spinal Cord (SC) tissue (8). Furthermore, damage to the Blood Spinal Cord Barrier (BSCB) allows neutrophils, in the first few hours postinjury, and macrophages, on the second- and third-days postinjury, to infiltrate the spinal cord at the injury site.

Consequently, a significant neuro-inflammatory process initiates the activation of the residing microglia. These inflammatory cells cooperate to remove the necrotic remains and the released myelin products from the damaged axons (9). However, the released cytokines, chemokines, and the activated complement cascade components trigger apoptosis in some of the surviving neurons and glial cells while recruiting and stimulating astrocytes to begin the process of scar formation (8). Over time, astrocytes secrete several types of proteoglycans such as Chondroitin Sulfate Proteoglycans, NG2 proteoglycan, Phosphacan, Brevican, Versican, and Neurocan (10). During the chronic phase of SCI, these substances form a physical and chemical barrier known as the glial scar, which inhibits axon regeneration. Another significant process that develops after injury is oxidative stress. The original traumatic insult initiates oxidative stress due to cellular releases of cytoplasmic components in addition to mitochondrial dysfunction. Oxidative stress continues to persist through the secondary phase, as the neuroinflammation is still prominent (11).

In the past two decades, studying the pathophysiology of SCI in the acute and subacute phases has become a major focus among practitioners in the field in order to understand the underlying mechanisms and provide targets for therapeutic strategies that would prevent further expansion of the injury and avoid the continued loss of neuronal functions (3). Moreover, the nature of these early phases determines many potential tools to predict not only the injury severity but also neurological recovery (12). Some of the major difficulties preventing the effective management of SCI are identifying non-invasive, practical, specific, and sensitive predictive measures to diagnose the severity and treatment of SCI. In the presence of effective diagnostic and predictive tests, different treatments such as surgical intervention would be more personalized to each patient, which ensures better evidence-based practices (13). In this comprehensive review of the literature, we searched the PubMed database with keywords “Biomarkers” and “Spinal Cord Injury” and screened the results. We prioritize discussing clinically significant and reliable biomarkers that have the potential to predict recovery after SCI, distinguish an array of severities, monitor complications, and estimate neurological and non-neurological prognoses. Informative biomarkers with these characteristics are of primary interest in SCI research. There are common biological phenomena that typically account for the appearance of biomarkers in SCI patients. The first biomarker origin is from the BSCB breach, where cellular components such as proteins, phospholipids, neurotransmitters, and metabolites leak from the injury site into the Cerebrospinal Fluid (CSF) and blood. These components are derived from the SC neurons, glial cells, or are factors in the glial scar formation process. The second important source of biomarkers are products of the neuroinflammatory processes or regenerative attempts that occur in the subacute or chronic phase. These include an increase in expression of proteins such as cytokines and growth factors (14). Moreover, there are some efforts made to profile the CSF and blood components at different timepoints postinjury. These efforts are aiming to study the metabolomics and proteomics of the spinal cord utilizing broad array techniques to increase the probability of finding sensible correlations between some of the studied components and the clinical progression of the injury (15, 16). It is noteworthy to highlight the shortage of human studies for many of the suggested potential biomarkers (13). Although animal experiments may show some significance regarding the correlation between the biomarkers and recovery status, the translation of utilizing these biomarkers for making sensitive and specific predictions might still be troublesome. These challenges can be attributed to the differences in the nature of SCI between humans and experimental animal models, as human SCIs usually present in variable severities and often in the context of polytrauma. Also, the animal spinal cord tissue has a relatively more straightforward neuronal structure and tends to have a different recovery curve following moderate to severe injuries (17). Therefore, it is always preferred to test the presence of such biomarkers in the human SCI population.

Some of the determinants of quality for studying biomarkers are the clinical tools used to follow the neurological recovery state. The American Spinal Injury Association (ASIA) Impairment Scale (AIS) is used to dictate and categorize progression of the injury (18). In addition, the timing at which the biomarkers are tested is critical, as some biomarkers are specific to certain phases of SCI and some are present in varying quantities at different timepoints postinjury. Another contributing factor to the specificity of the biomarkers is the cellular source. For example, inflammatory markers might not serve as a specific predictive tool in the presence of general inflammatory reactions in patients suffering from multiple traumas in addition to SCI, therefore some markers that serve as neuronal cytoskeletal elements such as Tau or Glial Fibrillary Acidic Protein (GFAP) may be more appealing (19). In this review, we divided our discussion into segments. The first part narrates the studies of potential biomarkers in SCI that are directly related to the injury and neurological loss, subdivided into structural and inflammatory sections. An overview of the biomarkers' studies discussed in the review and their results is shown in a summary table (Table 1).

**Table 1:** A summary of Spinal Cord Injury (SCI) biomarkers.

**Biomarker**	**Paper**	**Sample Source**	**Results**
NF-H	(20)	Human CSF	Mean levels were significantly higher in AIS A and B patients than in AIS C and D patients.
NF-L	(21)	Human CSF	Patients with complete motor loss showed higher levels compared to patients with incomplete motor loss.
NF-L	(22)	Human Serum	Elevated levels were associated with more severe SCI and poorer neurological prognosis.
pNF-H	(23)	Human Plasma	Increased levels were associated with increased axonal/neuronal disruption and differentiated severe, moderate, and mild SCI cases based on pNF-H concentration.
pNF-H	(24)	Rat Serum	Presence of pNF-H distinguished injured rats from non-injured rats.
pNF-H	(25)	Human Serum	Higher levels were associated with more severe SCI.
GFAP	(26)	Human CSF	At 24 h, levels of GFAP predicted future AIS graded injury severities as well as 6-month post injury segmental motor improvements.
GFAP	(21)	Human CSF	Patients with complete motor loss showed higher levels compared to patients with incomplete motor loss.
GFAP	(27)	Human CSF	Higher levels correlated with higher injury severities and predicted future neurological outcome.
GFAP	(20)	Human CSF	No significant correlations between AIS grade and injury severity.
GFAP	(25)	Human Serum	Higher levels were associated with more severe SCI.
NSE	(25)	Human Serum	Higher levels were associated with more severe SCI.
NSE	(20)	Human CSF	Mean levels were significantly higher in AIS A and B patients than in AIS C and D patients.
NSE	(28)	Rat Serum	Higher levels were associated with more severe SCI.
NSE	(29)	Rat CSF and Serum	Increased levels correlated with higher neurological defects and injury severity as well as increased in concentration in a stepwise manner to peak at 2 h postinjury.
S100-β	(29)	Rat CSF and Serum	Increased levels correlated with higher neurological defects and injury severity as well as increased in concentration in a stepwise manner to peak at 2 h postinjury.
S100-β	(20)	Human CSF	Mean levels were significantly higher in AIS A and B patients than in AIS C and D patients.
S100-β	(27)	Human CSF	Higher levels correlated with higher injury severities and predicted future neurological outcome.
S100-β	(28)	Rat Serum	No significant difference in concentrations of injured and non-injured rats.
Tau	(20)	Human CSF	No significant correlations between AIS grade and injury severity.
C-Tau	(27)	Human CSF	Higher levels correlated with higher injury severities and predicted future neurological outcome.
MAP2	(30)	Rat Dendrites	Presence of MAP2-immunoreactive dendrites extending into white matter with extensive beading patterns indicated worse behavioral recovery.
MBP	(31)	Mouse and Rat oligodendrocytes	Possible development of new myelin.
MBP	(32)	Swine CSF	Concentrations in injured swine were significantly higher than healthy controls. Injured swine MBP levels steadily increased over a 3-h period, possible indicating remyelination efforts.
MMPs	(33)	Human Serum	MMP-8 and 9 were upregulated post injury.
MMPs	(34)	Mouse and Rat CSF	Increased concentrations of MMP-8 correlated with poorer neurological recovery.
MMPs	(14)	Mouse and Rat CSF	Significant correlation between elevated MMP-9 levels and impaired neurological recovery.
MMPs	(35)	Canine CSF	MMP-9 levels 7 days postinjury were elevated in dogs that had the more severe IVDH injuries.
TGF-B	(36)	Human post-mortem spinal cord tissue	Injured tissue showed high levels of TGF-B1 two days postinjury, and TGF-B2 24 h postinjury.
TGF-B	(37)	Human Serum	An initial decrease in the concentrations of these cytokines was followed by a significant increase. 12 weeks postinjury, the observed elevated levels were correlated with the absence of neurological recovery.
IGF-1	(37)	Human Serum	An initial decrease in the concentrations of these cytokines was followed by a significant increase. 12 weeks postinjury, the observed elevated levels were correlated with the absence of neurological recovery.
IGF-1	(38)	Human Serum	Higher concentrations were correlated with greater neurological recovery.
sCD95L	(37)	Human Serum	An initial decrease in the concentrations of these cytokines was followed by a significant increase. 12 weeks postinjury, the observed elevated levels were correlated with the absence of neurological recovery.
sCD95L	(39)	Human Serum	Concentrations during the first week significantly decreased, followed by an increase in the second week, and reached peak expression during the 4th week post injury, possibly indicating the apoptotic effect to the spinal cord tissue.
sCD95L	(40)	Human Serum	Levels dropped at 4, 9, 12, and 24 h postinjury, and increased at 8 and 12 weeks.
sCD95L	(37)	Human Blood	High concentrations proved to be a marker for poor neurological improvement based on ASIA classification.
TNF-alpha	(41)	Human Serum	Patients who experienced an improvement in AIS grade also had a significant decrease of TNF-Alpha over 12 weeks.
TNF-alpha	(27)	Human CSF and Serum	Concentration predicted the AIS grade of the patient and the neurological recovery 6 months later.
TNF-alpha	(42)	Human CSF	No significant correlations were found between serum concentrations and injury severity and ASIA classification.
TNF-alpha	(43)	Human Serum	Increased concentrations were associated with an increased risk of NP
TNF-alpha	(44)	Rat and Mouse Serum	Elevated levels of IL-1B and IL-6 were found in injured rats when compared to non-injured controls.
ILs	(44)	Rat and Mouse Serum	Elevated levels of IL-1B and IL-6 were found in injured rats when compared to non-injured controls.
ILs	(27)	Human CSF	Higher levels of IL-6 and IL-8 correlated with higher injury severities and predicted future neurological outcome.
ILs	(42)	Human Serum	No associations between elevated serum concentration and injury degree were found. Alternatively, increased levels were correlated with injury complications i.e., NP, UTI, or pressure ulcers.
ILs	(41)	Human Serum	Concentrations between 4 h and 1-week postinjury did not correlate to any improvements or declines in neurological recovery. However, between 1 and 4 weeks it showed a significant drop exclusively in patients who experienced less improvement.
MCPs	(26)	Human CSF	24 h postinjury MCP-1 levels could predict patients' ASIA grade.
MCPs	(27)	Human CSF	Significantly lower MCP-1 concentrations in the patient groups that achieved improvement vs. those who did not.

The second portion reviews studies utilizing proteomics techniques to profile CSF or serum components after SCI. Lastly, we discuss the current status of biomarkers of SCI-related complications and what needs to be addressed in chronic SCI patient populations.

## Biomarkers Directly Related to Traumatic Spinal Cord Injury

### Structural Biomarkers

#### Neurofilament Proteins (NF)

Neurofilaments (NFs) are cytoskeletal proteins that are expressed abundantly and uniquely in the cytoplasm of axonal fibers in the CNS. NF regulates axonal transport and signaling (12), and has been a focus in neurological disorders due to the extracellular accumulations of NF in multiple neurological pathologies (45). NF can be divided into three major subunits: Neurofilament- light (NF-L), medium (NF-M), and heavy (NF-H) chains and are thought to be released from the cytoplasm of damaged neurons in traumatic SCI. Moreover, in the progression of secondary injury, as apoptosis and neuroinflammation are peaking, NF is thought to leak extracellularly along with other cytoplasmic components (45). Therefore, they are hypothesized to potentially indicate the severity of the neuronal loss in SCI as well as the extent of damage in the secondary stages.

Phosphorylated NF-H (pNF-H) is the primary subunit studied as a possible biomarker for CNS injury because of its traceable leakage into the CSF and serum following trauma (22, 24). A clinical study by Hayakawa et al. consisting of 14 patients with acute cervical SCI showed pNF-H levels in plasma correlated to injury severity. Enzyme-Linked Immunosorbent Assay (ELISA) revealed increased pNF-H was associated with increased axonal/neuronal disruption. pNF-H also successfully differentiated severe (grade A), moderate (grade B), and mild (grades C + D) cases, indicating it has the potential to accurately reflect different forms of SCI as well as severity (23).

In another study, Shaw et al. demonstrated the use of serum pNF-H as a distinguishing factor between injured and control rats. The model consisted of two injury groups; one received a contusion and the other a spinal cord hemisection injury. Both were compared to a sham group, which received only laminectomies. ELISA revealed high pNF-H levels were present only in the injury groups, while the sham groups remained negative (24).

In addition to pNF-H, NF-H has also commonly been examined as a potential SCI marker. A prospective cohort study measured CSF levels of NF-H along with other structural proteins (GFAP, neuron-specific enolase, S-100Beta, and tau) and were tested in 16 acute SCI patients within 24 h postinjury to be correlated with baseline AIS classifications and again at 6 or 12 months for further analysis of their release into the CSF. Mean NF-H levels were significantly higher in AIS A and B patients when compared to the levels in AIS C and D (20).

NF-L has also been studied as a potential biomarker for SCI. In one of the earliest clinical studies of SCI biomarkers, CSF samples were drawn from 6 acute SCI patients and were tested for NF-L and GFAP. Patients with complete motor loss showed higher levels of both biomarkers than patients with incomplete injury. In the same study, out of 17 patients with severe whiplash injuries, 3 patients had increased CSF levels of NF-L, indicating neural damage (21).

In another clinical study of patients with central cord syndrome (*n* = 4) and patients with varying degrees of motor loss SCI (*n* = 23) tested against healthy controls (*n* = 67), an Electrochemiluminescence (ECL) technique was used to quantify NF-L concentrations in serum. They found elevated levels of NF-L was associated with more severe SCI and poorer neurological prognosis (22). These results demonstrate the potential use of NFs as SCI biomarkers, despite the need for bigger sample sizes, more statistically significant correlations, and more specific clinical tools.

#### Glial Fibrillary Acidic Protein (GFAP)

GFAP is an intermediate filament protein found exclusively in the astroglia of the CNS. GFAP is responsible for the proper assembly and development of the cytoskeleton of astroglial cells, and upon injury or dysfunction, GFAP expression is upregulated by these cells (46). GFAP is an established biomarker for traumatic brain injury (TBI) and shows promise for similar applications in SCI (12, 47).

In the context of SCI, Kwon et al. studied GFAP and an array of cytokine concentrations in the CSF in a mix of 27 complete and incomplete SCI patients. The samples were taken within 72 h and analyzed using ELISA and multiplex cytokine array systems. At 24 h, levels of GFAP predicted, with 89% accuracy, future AIS graded injury severities as well as predicted 6-month postinjury segmental motor improvements (26). In a more recent study by Kwon et al. comprising of 50 acute SCI patients of varying severities scaled according to AIS, GFAP concentration in the CSF measured 24 h postinjury not only correlated with the severity of the injury but served as a predictor of future neurological outcome (27). GFAP concentrations differed significantly between patients that had improved recovery and those that did not (defined by a change in AIS grade and motor score) over the course of 6 months. An accuracy of 83% was achieved in predicting AIS outcome using linear discriminant analysis monitoring. These findings have important implications for identifying and predicting recovery in SCI patients. Another study corroborating these findings analyzed GFAP, pNF-H, and Neuron Specific Enolase (NSE) concentrations in 35 SCI patients of varying severities. GFAP serum levels were sampled at 24, 48, and 72-h timepoints postinjury and analyzed using ELISA. After 24 h, mean serum levels of GFAP in SCI patients were significantly higher than healthy control levels. Also, there was a significant variation of GFAP levels between grade A, grade B and grades C + D cases. Therefore, they concluded that GFAP at 24 h postinjury would be helpful to estimate the severity of SCI. They also attributed the drop in protein concentrations at 48 and 72 h postinjury to surgical decompression. However, if combined with neurological testing, they can offer more accurate estimates of SCI severity before spinal Computed Tomography (CT) or surgical interventions (25). In another study by Pouw et al. mentioned previously, 16 acute traumatic SCI patients' GFAP and other structural protein levels in the CSF were measured using sandwich ELISAs within 24 h after injury and tested against admission, 6 m, and 12 m AIS classifications. All protein concentrations were significantly elevated in SCI patients when compared to healthy controls. Although other proteins such as NSE and NFH concentrations significantly correlated with motor complete or incomplete functionality, GFAP showed no statistically significant correlations in this fashion, nor with AIS grade differentiation. One possible explanation for this, besides the small sample size, could be that GFAP does not reach peak plasma levels until after 24 h postinjury, which is outside the window tested in this study (20).

#### Cleaved Tau (C-Tau)

Tau is a Microtubule Associated Protein (MAP) that maintains the stabilization of axons and plays a role in axonal transport (12). After the injury, C-Tau is present in high concentrations within disrupted axons, and detectable in the sera/CSF upon BSCB breach (46). Kwon et al. studied Tau in addition to GFAP in CSF of SCI patients (referenced above) and found the same correlations of higher protein concentrations in more severe cases of SCI (27). As previously mentioned, Pouw et al. performed a study on 16 acute SCI patients with ranging severities on AIS and found that although there was a tendency of tau CSF concentrations to increase with increasing severity, there were no statistically significant differences between protein concentrations and AIS grades (20). These results are in direct contrast to the study performed by Kwon et al. and doubt the reliability of Tau as a SCI biomarker, although it could be due to a small sample size possibly. However, the study also concluded patients categorized into AIS A that contained lower concentrations of tau demonstrated higher incidences of conversion to AIS B (20). These discrepancies in the literature necessitate further research efforts to properly identify tau's potential role as a SCI biomarker.

#### S100-β

S100-β is a calcium binding protein found in glial cells and has previously been established as a marker for brain injury (12). S100-β has a wide variety of homeostatic activities including regulation of calcium fluxes, cell proliferation and differentiation, enzymatic/metabolic activity, and stabilization of MAPs (12, 46). S100-β is another structural marker reported to potentially predict SCI recovery in an aforementioned clinical study (27). Another experiment by Low et al. using an ELISA revealed increased S100-β serum levels 6 h after injury in 30 rats that underwent a contusive SCI when compared to control rats receiving only a laminectomy (28). However, 24 h after injury there were no significant differences in S100-βconcentrations between sham and injured rats. The number of studies available on S100-βlevels in tissue, serum or CSF is insufficient, and is worthy of further investigation in both animal models and the clinical level.

#### Neuron Specific Enolase (NSE)

NSE is an enzyme found primarily in neuronal cytoplasm, belonging to the glycolytic enzyme enolase family. NSE is an established biomarker for ischemic brain injury and axonal deformation and is released in high concentrations following damage to axons to reestablish cellular homeostasis (48). In an experimental rat model of SCI, NSE was abundantly expressed in the spinal cord tissue at the injury site in both neurons and glial cells, especially replicating microglia and astrocytes. These results indicated a significant link between NSE and neuroinflammation and astrogliosis after SCI (49). Because of these atypically high concentrations in surrounding tissues postinjury, NSE may serve as a useful biomarker. Loy et al. (discussed above) studied NSE in addition to S100-β and found significantly increased serum levels of NSE at both 6 h and 24 h when compared to sham animals (28). In a similar experimental study, serum and CSF levels of NSE along with S100-βwere measured in a rat acute SCI model at multiple timepoints postinjury (30 min, 2, 6, 12, and 24 h). The severity of the injury models correlated with the neurological deficits found later on, and the levels of both proteins in CSF and serum significantly correlated with the severity of the injury. Interestingly, both proteins increased in CSF and serum in a timely stepwise manner immediately after the injury to peak at approximately 2 h postinjury (29). Although the data about NSE shows promising potential to serve as a biomarker for SCI severity and to predict some prognostic outcomes, according to our literature survey, clinical studies are still lacking and essential in order prove these correlations and extrapolate any predictive use of NSE.

#### Microtubule-Associated Protein (MAP) 2

Microtubule-Associated Protein 2 (MAP2) is a protein specific to dendrites and has been previously used as a marker for dendritic injury (46). MAP2 utilizes a tubulin-binding domain to interact with the acidic portion of the C-terminal region found on tubulin (50). Although MAP2 has been routinely used as a TBI marker, there is an overall paucity of studies testing MAP2 in SCI. One study by Zhang et al. utilized a rat contusion injury model and found a rapid loss of MAP2 within 1–6 h. On the contrary, there was some correlation between MAP2-immunoreactive dendrites extending into the white matter displaying an extensive beading pattern and the behavioral recovery in the animals (30). Similarly, there is a shortage of studies (animal and human) to test the use of MAP2 as a cytoskeletal dendritic injury marker in the early phase of the injury.

#### Myelin Basic Protein (MBP)

Myelin Basic Protein (MBP) is found abundantly in the white matter; forming and maintaining the structure of the compact myelin sheath (46). MBP is comprised of positively charged amino acid groups and contains 4 primary isoforms. This heterogeneity is due to separate mRNA translation events (51). MBP is primarily studied in the context of TBI and Multiple Sclerosis (MS), however, due to its critical involvement in the myelination of axons, it has promise as a SCI biomarker. In one study, Hesp et al. demonstrated in a spinal cord contusion rat and mouse injury model that 3 months postinjury, immunostaining showed localization of MBP in newly formed oligodendrocytes, suggesting the development of new myelin (31). In another study aiming to simulate behind the armor blunt trauma to the spinal cord, pigs, wearing protective body armors, were shot in the T8 vertebrae. Tissue, CSF and serum levels of MBP were measured using an ELISA, which revealed that postinjury levels of MBP in the CSF steadily increased over a 3-h period. Additionally, MBP concentrations in injured swine were significantly higher when compared to healthy controls (32). Therefore, MBP is thought to represent a potential indicator of remyelination efforts in the process of recovery after SCI.

#### Matrix Metalloproteinases (MMPs)

Matrix Metalloproteinases (MMPs) are 23 different isoforms, which work in tandem with neurons and glia to modulate cellular migration via the degradation of extracellular matrix (ECM) proteins. They are critical to the CNS's injury response regulation repair (52). Popular methods in the literature to quantify MMP expression are Polymerase Chain Reaction (PCR), western blots, ELISA, Immunohistochemistry (IHC), gelatin zymography, and enzyme activity assays (52). MMPs are expressed at the highest rates during the subacute phase of SCI, indicating a potential to serve as a possible biomarker for predicting the future neurological outcome (53). In a recent clinical study, Moghaddam et al. analyzed the presence of MMP-2, 8, 9, 10, and 12 in the serum of 115 patients with traumatic SCI over the course of 12 weeks. Using a High Sensitivity Cytokine Panel, they found multiple forms of MMP were upregulated, specifically MMP-8 and 9, which they concluded could be a useful indicator for recovery potential (33). Light et al. utilized a rodent SCI model where MMP-8 levels were analyzed 12 days postinjury in the CSF using a 34-cytokine sandwich microarray. The results showed increased concentrations of MMP-8 in rodents that correlated with poor neurological recovery (34). In corroboration of this finding, Kwon et al. performed a similar rodent study evaluating CSF levels of MMP-9 and other possible biomarkers over a 7-day time course postinjury. They found a significant correlation between elevated MMP-9 levels and impaired neurological recovery (14). Another study analyzing CSF levels of MMP-9 in canines with an Intervertebral Disc Herniation (IVDH) injury model, 6 dogs with variable degrees of neurological deficits after IVDH were included. MMP-9 levels 7 days postinjury were markedly elevated in animals that had the most severe injuries and worst prognostic profile (35). These studies provide some evidence that MMPs are a good candidate for biomarkers in delayed phases after acute SCI, which may suit some clinical scenarios.

### Inflammatory Biomarkers

#### Transforming Growth Factor Beta (TGF-B)

TGF-B is a polypeptide that regulates a wide variety of biological functions including stem cell differentiation, recovery processes, inflammation, and immune responses, and embryogenesis. The mechanism by which TGF-B acts is through transmembrane kinase receptors serine and threonine (54), as well as by astrocytic phosphorylation of Smad2. Ultimately, TGF-B hinders neurite extension, promotes astrogliosis, and accumulation of proteoglycans (55). Subunits of TGF-B, 2 and 3, are present ubiquitously in the CNS, while TGF-B1 is predominantly found in portions of the basal ganglia, cerebral cortex, choroid plexus, and meninges (56). To better understand TGF-B1 and 2 in SCI, Buss et al. analyzed the expression of these subunits post-mortem in the spinal cord of patients with SCI. When compared to healthy controls, injured tissue showed high levels of TGF-B1 2 days postinjury, and TGF-B2 24 h postinjury, which retained its abnormally high levels for 1 year. While it seems TGF-B1 and 2 play a critical role in the subacute inflammatory response, in order to verify these findings, a larger sample size is necessary (36). Additionally, a study by Schachtrup et al. identified that TGF-B is carried by blood protein fibrinogen, allowing for its easy accessibility upon upregulation (55). Ferbert et al. conducted a clinical study measuring TGF-B1, Insulin-like growth factor 1 (IGF-1), and sCD95L in the serum of 23 SCI patients. In the acute and subacute injury phase, an initial decrease in the concentrations of these cytokines was followed by a significant increase. Twelve weeks postinjury, the observed elevated levels were correlated with the absence of neurological recovery, implying the potential use of these cytokines to predict the progression of SCI (37).

#### Tumor Necrosis Factor (TNF)

TNF-Alpha is a proinflammatory cytokine expressed within a few hours after injury, suggesting it may be a useful indicator of SCI pathology (57). Upon injury, the BSCB is often breached, allowing for the migration of leukocytes and other cells into the spinal cord. After trauma, these cells, along with the resident microglia, secrete TNF-Alpha, which adds to the overall inflammatory stress of the injury (12). Biglari et al. measured TNF-Alpha and Interleukin 1 (IL-1) concentrations in the serum of 23 SCI patients over a 12-week period post-injury. Patients who experienced an improvement in AIS also had a significant decrease of TNF-Alpha (41). In another clinical study, Kwon et al. analyzed TNF-Alpha levels using a multiplex cytokine array system and standard ELISA in the CSF and serum of 27 patients with complete SCI. These measurements, taken at 24 h post-injury, predicted with 89% accuracy the AIS grade of the patient as well as the neurological recovery 6 months later (27).

On the contrary, Davies et al. measured TNF-Alpha in a clinical study consisting of 56 patients with SCI of different degrees of severity with matched controls. No significant correlations were found between serum concentrations and injury severity and ASIA classification (42). These results suggest that CSF samples are more reliable for predicting neurological recovery and injury severity (14, 58). However, serum concentrations were found to be useful in predicting the onset of specific complications such as neuropathic pain (NP), urinary tract infection (UTI), and pressure ulcers. TNF-alpha is one example that is suggested as a predictive tool for several long-term complications of SCI. Xu et al. examined the relationship between inflammatory markers such as TNF-Alpha and NP in 70 chronic SCI patients. They found increased concentrations of this cytokine in the serum was associated with an increased risk of NP, thereby concluding TNF-Alpha has the potential to predict chronic NP in SCI patients (43). These data need to be interpretted with caution. Cytokine levels in serum generally spike due to systemic inflammation in response to trauma, and may not be the most specific indicator of inflammation in SCI. As a result, it is recommended that they be used in parallel with other diagnostic biomarkers to ensure accuracy (12). Interestingly, Neefkes-Zonneveld et al. performed a systematic review of the relationship between long-term physical therapy and the levels of inflammatory markers in several human studies. They found that improvement was correlated with a decrease in expression of these serum inflammatory markers (59). In another review, the levels of serum adipokines such as TNF-Alpha dropped with exercise, and was attributed to the fact that it is released by adipose tissue, concluding that exercise can help with decreasing the general systemic inflammation state in our patient population (60, 61). These results indicate the influence of factors like exercise on the serum levels of inflammatory cytokines. Therefore, CSF appears to be a better source for sampling inflammatory markers in a SCI-specific context when compared to their serum levels, as they were found much lower in comparison to their levels in the spinal cord tissue (14, 58).

#### Insulin-Like Growth Factor (IGF)

IGF−1, also known as Somatomedin C (SM-C), is a soluble growth factor primarily made in the liver that is expressed in myocytes, bone cells, chondrocytes, and other various tissues. It contains 70 amino acid peptide chains and has multiple traceable isoforms that can be found in blood serum (38, 62). In an *in vitro* model, IGF-1 demonstrated the ability to increase the survival of neuronal cells and impede excitotoxicity in motor neurons (62, 63). In a clinical study consisting of 45 traumatic SCI patients, IGF-1 was measured in the peripheral blood serum 1-week postinjury and revealed that higher concentrations of this growth factor were correlated with greater neurological recovery (38). In contrast, the study performed by Ferbert et al. IGF-1, TGF-Beta, and Soluble CD95 Ligand (sCD95L) levels showed a significant increase in their serum levels in patients with worse neurologic recovery 12 weeks postinjury (37). Because of the discrepancies found in the literature, there is a need for better designed human studies with bigger sample sizes to examine the fluctuation of its serum levels, possibly using its levels as an adjunct to other biomarkers to achieve the best predictive correlations with neurological recovery.

#### Interleukins (ILs)

Interleukins (ILs) are a family of cytokines produced by leukocytes that help regulate and stimulate immune function and growth. The proinflammatory role of ILs in SCI has been previously well-characterized in the literature. For example, Wang et al. identified Interleukin 1 beta (IL-1B) as a critical player in increasing inflammation and glial scar tissue formation in SCI (64). Hasturk et al. corroborated this finding in their study, which revealed elevated serum levels of IL-1B, IL-6, and TNF-Alpha in a rodent ischemia/reperfusion injury model when compared to controls (44).

In the study performed by Biglari et al. (previously mentioned) following the temporal changes in the serum levels of IL-1B and TNF-Alpha in 23 SCI patients, IL-1B concentrations fluctuated greatly between 4 h and 1-week post-injury. During this time period, the differences between these levels did not correlate to any improvements or declines in neurological recovery. However, between 1 and 4 weeks postinjury, IL-1B showed a significant drop exclusively in patients who experienced less improvement. Also in this study, patients received neither corticosteroids nor non-steroidal anti-inflammatory medications which offers a good chance to extrapolate their results (41). Additionally, Kwon et al. included reports on IL-6 and IL-8 in their cohort of 50 SCI patients. As previously discussed, the CSF levels of these cytokines were significantly different, and predicted with 89% accuracy patient improvements of an AIS grade over 6 months after injury compared to those that did not improve. Additionally, 6-month motor score improvement was correlated with these cytokines' levels 24 h postinjury. Specifically, IL-6 and S100B CSF levels 24 h postinjury correlated with the conversion from AIS A to B or C (27). These studies provide evidence that ILs are strong candidates for inflammation biomarkers in the context of SCI.

However, a study performed by Davies et al. analyzing IL-1B, 2, 4, and 6 levels in the serum of 56 SCI patients with varying severities demonstrated no associations between elevated ILs serum concentrations and injury degree. Alternatively, increased levels of ILs were correlated with subjects who presented complications of the injury, i.e., NP, UTI, or pressure ulcers. Although this aligns with the nature of inflammatory cytokines in the systemic circulation, the presence of these cytokines might not necessarily be indicative of injury severity, especially if the patient has progressed passed the acute/subacute phase and has entered chronic SCI stage. However, in general, these conclusions still suggest ILs as a predictor of inflammatory-related pathologies in SCI, which can result in improved treatments for these conditions (42).

#### Soluble CD95 Ligand (sCD95L)

sCD95L, also known as the Fas ligand, plays a critical role in the induction of the extrinsic apoptotic cascade, which is a vital portion of the pathophysiology in the subacute stages of SCI. Following cleavage of type II transmembrane protein CD95L, sCD95L is released, and binds to Fas to activate apoptotic pathways (65, 66). sCD95L regulates activation-induced cell death, and therefore plays an important role in maintaining multiple immune functions as well as in cancer stem cells survival (67). Studies have shown that after spinal cord injury, CD95 receptor production is upregulated on the oligodendrocytes and spinal cord neurons, leading to activation of the apoptotic cascades in these cells, and a further loss of spinal cord cell population after the primary trauma (68, 69). As CD95L is cleaved and released, a portion of it extravasates to the peripheral blood, facilitated by the breach in BSCB, which might explain the changes in its levels in the serum, especially at the subacute phases when apoptotic activity is more prominent (70). Also, several preclinical studies have demonstrated that CD95 deficient mice, targeting CD95 receptors, or using sCD95R intrathecally, to neutralize sCD95L in CSF, could reduce apoptosis, tissue destruction and achieve better functional recovery (71–73). These results provide a rationale for testing sCD95L serum/CSF levels as a potential biomarker in the context of SCI.

Biglari et al. conducted a pilot study in 8 SCI patients analyzing serum sCD95L levels using ELISA. Samples from the patients were collected on the 1st and 3rd days of admission and in the 1st, 2nd, 4th, 8th, and 12th weeks after injury. The serum concentrations during the first week significantly decreased, followed by an increase in the second week, and reached peak expression during the 4th week in all 8 subjects. Due to the study's small sample size, it was difficult to draw meaningful conclusions, however, sCD95L appears to have potential to serve as a biomarker in the subacute stage of SCI, particularly, to reflect the destructive apoptotic effect to the spinal cord tissue and the subsequent neurological loss (39). As an extension, Biglari et al. confirmed serum levels of sCD95L in 23 SCI patients dropped at 4, 9, 12, and 24 h postinjury, while levels increased at 8 and 12 weeks (40). Although the study provided significant differences in the levels hours after injury vs. 8–12 weeks after injury, the study failed to present a healthy control group lacking SCI. In a following study by the same group, Ferbert et al. evaluated sCD95L levels in blood samples from 23 SCI patients and their relationship to neurological outcome based on ASIA classification. Significantly high sCD95L serum concentrations proved to be a marker for poor neurological improvement, demonstrating sCD95L's potential as a SCI biomarker (37). In summary, sCD95L has consistently shown CSF level patterns which can serve as a therapeutic target as well as a specific marker in the secondary phases of injury, particularly apoptosis (12).

#### Growth Factors

Although insufficient work has been invested in linking different types of CNS growth factors to SCI in the context of diagnostic measures, plenty of studies highlighting their regenerative potentials have been discussed. Some of these growth factors include Brain-derived Neurotrophic factor (BDNF), Glial cell-derived Neurotrophic Factor (GDNF), Neurotrophin-3, and Neurotrophin-4/5 (74). Moreover, Nerve Growth Factor (NGF) was investigated for its potential benefit in monitoring lower urinary tract dysfunction (mentioned later in complications section). We hypothesize that these growth factors can act as biomarkers for SCI in serum and/or CSF which should be studied in more depth, due to the therapeutic success of these growth factors in the previous literature (74).

#### Macrophage Inflammatory Proteins (MIPs) and Monocyte Chemoattractant Proteins (MCPs)

MCP-1 and MIP-1alpha are chemotactic cytokines (chemokines) that are expressed in the spinal cord following initiation of the secondary phase of SCI and during the start of axonal degenerative processes (75). Although the function of these chemokines in SCI is unclear, they are thought to play a vital role in apoptosis and inflammation, as well as regulate clearance of cellular debris and released myelin, which are considered some of the critical factors that affect regenerative efforts by neurons and glial cells later on McTigue et al. (76), Zhang et al. (77), Perrin et al. (78), and Ousman and David (79). In a study by Kwon et al. MCP-1 was tracked along with other cytokines in the CSF of 27 complete SCI patients with variable ASIA classifications over 72 h postinjury. Their MCP-1 levels showed severity-related elevations. Moreover, the 24 h post-injury levels could predict patients' ASIA grade with relatively high accuracy, suggesting these cytokines as a potential tool for severity prediction for acutely injured patients (26). In a subsequent study by the same group, a larger patient population of 50 SCI patients presenting with varying AIS grades was included. CSF samples at 24 h postinjury were tested for ILs, S100B, tau, GFAP, and MCP-1 and they collectively showed again a significant difference in the patient groups that achieved improvement vs. those who did not. This provides increasing evidence of the potential benefits of these proteins as biomarkers (27). However, more human studies are required that include healthy control subjects to rule out analytical errors.

## SCI Biomarker Discovery Using Proteomics

With the recent emergence of new techniques to study proteome changes, proteomics is becoming an important avenue for biomarker discovery in SCI patients. Biomarker discovery using proteomics is generally done using a “shotgun” approach, where protein quantities are assessed using mass spectrometry or microarray-based techniques often combined with gel electrophoresis of tissue or CSF samples taken from SCI patients and compared to control groups (80, 81). This approach, therefore, removes bias and uncovers targets that describe the activity of various molecular pathways activated or suppressed after injury, effectively providing a temporal map of physiological response to injury that can be used to predict recovery and outcomes.

Recently, Moghieb A. and colleagues used reversed-phase liquid chromatography-tandem mass spectrometry was combined with immunoblotting and analyzed proteome changes within spinal cord segments caudal to the injury site at 24 h and 7 days following moderate or severe thoracic SCI in rats. Proteome analysis revealed upregulation of 22 proteins at both 24 h and 7 days post-SCI, as well as downregulation of 19 and 16 proteins at 24 h and 7 days, respectively. Further analysis identified 12 proteins as potential SCI markers: TF (Transferrin), FASN (Fatty acid synthase), NME1 (Nucleoside diphosphate kinase 1), STMN1 (Stathmin 1), EEF2 (Eukaryotic translation elongation factor 2), CTSD (Cathepsin D), ANXA1 (Annexin A1), ANXA2 (Annexin 2), PGM1 (Phosphoglucomutase 1), PEA15 (Phosphoprotein enriched in astrocytes 15), GOT2 (Glutamic-oxaloacetic transaminase 2), and TPI-1 (Triosephosphate isomerase 1). Out of these 12 potential biomarkers, TF, CTSD, TPI-1, and PEA15 were verified in both rat spinal cord tissue and CSF following SCI, as well as human CSF from SCI patients, therefore showing their potential use as biomarkers (82). Another study, led by Sengupta and colleagues, used difference gel electrophoresis, matrix-assisted laser desorption/ionization mass spectrometry, as well as immunoblotting, and analyzed 49 proteins from the CSF of SCI patients and identified a subset of 8 proteins of interest. Out of the 49 proteins analyzed in this study TF, Beta-2 glycoprotein I precursor, General transcription factor 2C polypeptide 5, Immunoglobulin gamma-4 chain C region, Immunoglobulin gamma-2 chain C region, and Zinc alpha 2 glycoprotein were abundant at 1–8 days post complete injury, while Haptoglobin, serum albumin precursor, and Transferrin were abundant only following incomplete injury at 1–8 days. This group also reported reversal in Haptoglobin and Zinc alpha 2 glycoprotein expression at 15–60 days postinjury both in incomplete and complete injury (16). Effectively, this report provides an additional set of protein markers for SCI progression and recovery potential.

Finally, several reports focused on matrix molecules responsible for ECM remodeling after injury, which is a critical part of SCI response and lesion formation. Proteome analysis revealed upregulation of various danger-associated molecular patterns, or known as alarmins, involved in the inflammatory response (83). One of the upregulated proteins involved in the inflammatory response was matrix metalloproteinase 8 (MMP-8), a neutrophil collagenase, which has been shown to correlate with tissue damage and BSCB disruption at subacute timepoints after SCI (34). Using mass spectrometry Didangelos and colleagues described a set of 47 alarmins upregulated at 8 weeks following T10 contusion in rats, and Western blot analysis confirmed upregulation of Asporin, Col1a1, Dermatopontin, Mimecan, Fibromodulin, Periostin, Prolargin, Decorin, and Neurocan after injury, while NF200 and Aggrecan were downregulated in injured samples. These alarmins are responsible for the inflammatory response and were found to act via IL1 and The nuclear factor (NF)-κB transcription factor (NFκB) signaling as well as toll-like receptor-4 (TLR-4) and the receptor for advanced glycation end-products (RAGE) (83).

## Biomarkers Role in Predicting and Managing SCI-Related Complications

In this section, we discuss biomarkers that are studied as potential predictive tools to help manage SCI-related complications such as pressure ulcers, NP, and urinary complications. The patient populations found in this section are mainly chronic SCI patients (estimated by 280,000 patients in the USA alone) with established spinal cord tissue pathology and neurological losses. Thus far, the primary attention is given to validating interventions to target neurological loss, preventing the progression of the SCI itself, stabilizing the patients, and preventing the consequent complications. We believe developing reliable biomarkers specific to certain complications is of high importance, as it will serve the chronic SCI patients and help them resume their social and daily activities, improve their quality of life, and reduce the utilization of healthcare resources.

### Pressure Ulcers

Pressure ulcers are considered a severe and prevalent complication for patients with chronic SCI, as some reports estimate the lifetime risk for these individuals at approximately 85% and recurrence rate that reaches up to 91% (84, 85). Consequently, such problems form a heavy burden on healthcare resources by adding up to the cost of continuous care for these patients and worsening their quality of life (86). Pressure ulcers commonly occur in the body parts affected by SCI, most commonly ischium, due to the loss of the natural skin's vital protective properties. The risk of their occurrence increases with the duration of the injury, age, lack of care, urinary incontinence, smoking and associated medical conditions such as diabetes mellitus (87). To understand the predisposing factors of pressure ulcers, reports attribute the pathophysiology of pressure ulcers to poor wound healing and diminished expression of leukocytic adhesion molecules (88). In one study, samples were taken from chronic SCI patients, and immunostained for several adhesion molecules typically expressed on leukocytes in peripheral blood and compared to samples from healthy controls. The results demonstrated a marked reduction of fibronectin expression in the ulcers, which led to poor leukocytic adhesion and interaction at the sites of the ulcers (89). Because the goal is to find reliable and practical biomarkers to predict and help prevent ulcers, few trials have been designed to test proteins, mainly inflammation-related, as potential candidates. In one study, whole blood and serum of SCI patients with pressure ulcers were compared to samples of ulcer-free SCI patients. Patients who presented with pressure ulcers demonstrated a significant correlation between levels/counts of C-reactive Protein (CRP), Hematocrit (Hct), lymphocytes, Red Blood Cells (RBCs), White Blood Cells (WBCs), and serum proteins and the grade of the pressure ulcers (90). In another study, ulcer and plasma or urine samples were taken from 32 individuals with chronic SCI and matched to urine or plasma samples from SCI patients with no ulcers. There was a statistical correlation between Interferon (IFN)-Gamma-induced protein in plasma as well as the drop of IFN-Alpha in urine and the occurrence if the first ulcer after SCI. This suggests that the changes of IFNs might be of use to predict pressure ulcers (91). However, we think markers might not carry a significant specificity to pressure ulcers and can be altered by several other systemic inflammation factors such as the high incidence of pneumonia and urinary tract infection in our population of interest (92). Therefore, further and larger human studies are required in addition to a broader spectrum of screening for new candidates (not only the hypothesis-driven ones), with the inclusion of healthy controls and pressure ulcer-free SCI patients.

### Lower Urinary Tract Dysfunction

Lastly, urinary tract dysfunction is considered one of the most impactful complications of SCI, as almost all patients suffer from some degree of urinary dysfunction postinjury. Some studies indicate about 80% of SCI patients reported urinary complaints as a major cause of reducing their quality of life (93). Such a persistent complication affects the patients' personal and social lives and is always one of the most distressing issues in health questionnaires. It also has negative psychological impacts, as it causes embarrassment, which can lead to a withdrawal from their community and the avoidance of physiotherapy and medical follow-up appointments (94). Briefly, immediately after SCI in the phase of the spinal shock, the urinary bladder becomes areflexic, causing patients to suffer from urinary retention. Following the resolution of spinal shock, lower urinary tract shows one of two main classic presentations based on the extent and the level of SCI. If the SCI is above the lumbosacral segment, it leads to loss of upper control on the sacral circuit preserving some of the sensory fibers, and after a while, reorganization of the sacral center occurs, leading to involuntary bladder contractions known as neurogenic detrusor overactivity (NDO). In addition, when involuntary micturition reflex is initiated, the synchronization between the detrusor contraction and the sphincter relaxation is lost, leading to detrusor-sphincter dyssynergia (DSD). This presentation leads to persistent high pressure in the urinary bladder, hence long-term deterioration of renal functions (95, 96). The other presentation occurs when the SCI is in the sacral segment or below, damaging the sacral micturition center and leading to loss of control of the detrusor and sphincter muscles as well as bladder sensation. Consequently, the urinary bladder becomes atonic and patients suffer from low-pressure overflow urinary incontinence, which increases the risk of urinary tract infections, but not deterioration of renal function (97). Currently, the management of this problem is using intermittent self-catheterization to relieve the high pressure and decrease its effect on renal functions (95). Pharmacological treatments such as intravesical irrigation with antimuscarinic drugs or sphincter injections with botulinum toxin have been introduced as well as implantation for artificial urinary sphincter to control the overflow incontinence (98, 99). Such interventions could achieve a better prognosis for SCI patients, yet the follow up for lower urinary tract functions requires regular urodynamic testing, including electromyographic recording for the external sphincter and radiologic examinations. These extra examinations complicate regular health care for patients, especially when they suffer from significant functional loss, and requires high and persistent will to commit to their medical care, thus the need for an easier, more practical tool to monitor the morphological and functional changes in the lower urinary tract and make the follow-up process more bearable (93). An additional benefit of any potential biomarker for urinary dysfunction would enable researchers to evaluate the effectiveness of pharmacological and surgical interventions. One protein that has been suggested as a candidate is NGF which is primarily secreted by urinary bladder mesothelium and transported in a retrograde fashion to sacral micturition centers. A study tested urinary levels of NGF in SCI patients and compared it to control adults and found a significant elevation of NGF in the urine of SCI patients who had elevated intravesical pressure due to NDO. Also, urinary NGF/Creatinine ratio showed a difference between urinary retention states after catheterization and relief of increased pressure, which indicates the sensitivity of urinary NGF levels in regards to monitoring renal changes related to NDO (100). In another clinical study, tissue NGF from endoscopic biopsies was used as a marker for recovery of a sustained increase in the intravesical pressure and compared to urodynamic recordings in 23 patients with NDO who were treated with intravesical Botulinum-A toxin (BTX-A) injections. BTX-A induced a state of NGF deprivation in the bladder tissue that lasted for up to 3 months (101). NGF reduction was also correlated with the drop of pressure resulting from detrusor relaxation, which indicates the strong link between the prolonged high intravesical pressure states and levels of NGF, either in tissue or urine, thus suggesting its link with SCI-related lower urinary tract dysfunction and the feasibility to use it as a monitoring tool (100). Interestingly, in another study analyzing Never Growth Factor (NGF) concentrations in the urine of 37 chronic SCI patients suffering from lower urinary tract dysfunction and 10 controls, there was no correlation between NGF levels and injury-related neurogenic lower urinary dysfunction. Factors that could have affected the outcome of this study include sample size, and patient injury severities (102). Such controversy requires NGF need to be studied on a bigger scale in human cohorts at different time points in the chronic phase and to be compared to healthy control levels to prove its sensitivity as a biomarker.

## Conclusion

For the past two decades, there has been a growing interest in developing novel, reliable, and practical tools to diagnose severity and predict the progression of SCI. The need for such tools is multidisciplinary. First, medical care decisions would become more personal and tailored to each case, which minimizes unnecessary interventions and makes patient follow-up easier. Secondly, such tools would be helpful in providing health care for SCI patients in developing countries that lack sophisticated medical resources. Third, it would substantially boost SCI research efforts at both preclinical and clinical levels. Possessing the tools to monitor specific changes related to the pathology of SCI would enable researchers to test more therapeutic strategies and increase the chances of improving the current medical practice. Although initial steps have been taken to research the use of biomarkers in SCI, additional efforts in this field are required before achieving approval from clinicians and surgeons to integrate them into everyday practice. Currently, there is a paucity of clinical studies showing high evidence for correlations between biomarkers and severity or improvement. One of the problems with studying biomarkers in CSF is the difficulty to obtain CSF sample especially from SCI patients as it is painful and is associated with a risk of epidural hematoma that may lead to further spinal cord compression.

Hypothesis-driven SCI biomarker studies are usually generated in an unintentionally biased process in which the researchers base their studies on proteins commonly reported to have major roles in the pathophysiology of SCI. This might lead to overlooking significant potential biomarkers if they are not given enough attention in the SCI literature. Thankfully, several research groups started to adopt broader approaches to quantify potential markers in CSF and serum samples using high throughput techniques to screen for the proteomic profiles at several time points after the injury. Although these studies are still sparse and often do not get translated to human trials, we think they offer great promise to uncover innovative and more specific biomarkers. We also suggest that biomarker studies put more focus on investigating postinjury correlations between the biomarkers of interest and imaging findings at later time points. Doing so may illuminate new links between structural or inflammatory proteins and MRI spinal cord tissue findings. Finally, one of the least researched areas in the field of SCI is identifying SCI-related complication biomarkers. Complications such as urinary bladder dysfunction and pressure ulcers cause significant distress to chronic SCI patients and can negatively affect all aspects of their lifestyle. Additionally, they require consistent and exhausting measures to follow up treatments and management, yet very few studies are targeting these biomarkers that can ease the process of follow-up and prediction. Ironically, there are at least 10 times more chronic SCI patients than new SCI cases, so, we hope these large numbers of patients are incorporated when designing clinical studies for SCI-related complications, as it will benefit SCI universally.

In conclusion, the biomarkers mentioned in this review were characterized based on their purpose in the direct neurologic insult of SCI, including those related to degrees of severity, recovery trajectory, and the occurrence of complications as a side effect of the initial injury. We prioritized presenting studies that were recent, contained practical methods of biomarker level validation, comprised of human subjects, and had a large sample size. Despite the growing popularity of the SCI biomarker field, more studies are required before we can integrate these marking techniques into the universal SCI screening and diagnostic standard of care. SCI biomarkers have the potential to serve as predictive measures for injury severity and neurological progression, as well as identify and alleviate complications of SCI, ultimately resulting in betterment of quality of life for patients that suffer from SCI.

## Author Contributions

AA: study design, data collection, writing, and submission; AR and PS: data collection and writing; SA, EE, and NO: data collection; DS, AO, and BA: study outline, reviewing- proofreading, and formatting.

### Conflict of Interest Statement

The authors declare that the research was conducted in the absence of any commercial or financial relationships that could be construed as a potential conflict of interest.
